# Kv7 channels are upregulated during striatal neuron development and promote maturation of human iPSC-derived neurons

**DOI:** 10.1007/s00424-018-2155-7

**Published:** 2018-05-24

**Authors:** Vsevolod Telezhkin, Marco Straccia, Polina Yarova, Monica Pardo, Sun Yung, Ngoc-Nga Vinh, Jane M. Hancock, Gerardo Garcia-Diaz Barriga, David A. Brown, Anne E. Rosser, Jonathan T. Brown, Josep M. Canals, Andrew D. Randall, Nicholas D. Allen, Paul J. Kemp

**Affiliations:** 1 School of Dental Sciences, Newcastle University, Framlington Place, Newcastle upon Tyne, NE2 4BW UK; 2 School of Biosciences, Cardiff University, The Sir Martin Evans Building, Museum Avenue, Cardiff, CF10 3AX UK; 3 Department of Neuroscience, Physiology and Pharmacology, London University College, London, UK; 4 Department of Cell Biology, Immunology and Neuroscience, Faculty of Medicine, August Pi Sunyer Biomedical Research Institute (IDIBAPS), University of Barcelona, Barcelona, Spain; 5 Centro de Investigación Biomédica en Red sobre Enfermedades Neurodegenerativas (CIBERNED), Barcelona, Spain; 6 School of Physiology and Pharmacology, University of Bristol, Bristol, UK; 7 Institute of Psychological Medicine and Clinical Neurosciences, School of Medicine, Cardiff University, Hadyn Ellis Building, Cardiff, CF24 4HQ UK; 8 Hatherly Laboratory, Institute of Biomedical and Clinical Sciences, University of Exeter Medical School, Exeter, UK

**Keywords:** *KCNQ*, Kv7, Human-induced pluripotent stem cells, Striatum, Patch-clamp

## Abstract

Kv7 channels determine the resting membrane potential of neurons and regulate their excitability. Even though dysfunction of Kv7 channels has been linked to several debilitating childhood neuronal disorders, the ontogeny of the constituent genes, which encode Kv7 channels (*KNCQ*), and expression of their subunits have been largely unexplored. Here, we show that developmentally regulated expression of specific *KCNQ* mRNA and Kv7 channel subunits in mouse and human striatum is crucial to the functional maturation of mouse striatal neurons and human-induced pluripotent stem cell-derived neurons. This demonstrates their pivotal role in normal development and maturation, the knowledge of which can now be harnessed to synchronise and accelerate neuronal differentiation of stem cell-derived neurons, enhancing their utility for disease modelling and drug discovery.

## Introduction

Kv7 channels [4] are sub-threshold, voltage-gated potassium ion channels that are widely expressed in mammalian central and peripheral neurons [5]. They were first described in sympathetic neurons [4] and have subsequently been identified in a variety of neurons including medium spiny neurons (MSNs) of the striatum [33], sensory nociceptive structures [6, 27], hippocampal CA1 pyramidal cells [14, 31, 32], as well as in the substantia nigra [34]. Neuronal Kv7 channels are assembled of two Kv7.2 and two Kv7.3 subunits, most commonly, but not invariably [18, 40]. Functionally, they help to maintain and stabilise the neuronal resting membrane potential (Vm), thereby controlling neuronal excitability and action potential threshold [11]. In mammals, Kv7 channels, and/or their constituent subunits, are developmentally upregulated during early post-natal life [13, 38, 41], with associated changes in Vm [23] and excitability [30]. Kv7 channels have also been reported to play roles in survival, maturation and synapse development in embryonic stem cell-derived and human-induced pluripotent stem cell (hiPSC)-derived neurons [35, 44]. Being a critical determinant of neuronal excitability, regulated expression of Kv7 channels during neuronal differentiation and maturation ought logically to be vital for the progressive hyperpolarization of Vm and its stabilisation below the threshold for voltage-gated Na^+^ channel activation/inactivation [5], during early development. However, the importance of Kv7 channels in pre-natal neuronal development and maturation remains largely unexplored. In the present experiments, we have considered how far the developmental expression of Kv7 channels might contribute to the functional maturation of striatal neurons. Within the striatum, mature MSNs play an important role in the control of movement and in a range of cognitive and behavioural functions [7, 12] and show prominent degeneration in Huntington’s disease (HD) [39]. They display conspicuous Kv7 currents which contribute to their Vm and action potential threshold to regulate their excitable behaviour [8, 33].

Thus, the present study was designed to test firstly the hypothesis that the functional neuronal development of mouse embryonic striatal neurons, isolated at embryonic day (E)13, E15 and E17 and cultured in vitro for up to 16 days, is associated with enhanced expression and activity of Kv7 channels. Once this was established in mouse striatum, the ontogenies of Kv7 subunits were determined in developing and adult striatum of the human brain. We then investigated the functional and molecular expression of Kv7 channels during neuronal differentiation and maturation of hiPSC-derived neurons. Finally, the idea that forced co-expression of Kv7.2 and Kv7.3 might represent a novel method by which to accelerate the functional maturation of neurons in vitro was tested using two independent hiPSC lines. Taken together, these data show that progressive expression of Kv7 channels during development of striatal neurons leads to hyperpolarization and increased excitability in vivo and that forced expression of Kv7 channels results in enhanced functional maturation of stem cell-derived neurons in vitro*.*

## Methods

### Isolation and culture of embryonic mouse striatal neurons

Striata were dissected from mouse embryos at stages E13, E15 and E17 and placed into hibernate medium (Invitrogen Life Technologies, Paisley, Strathclyde, UK), gently triturated using a P1000 pipette and then centrifuged at 1000 rpm for 2 min. The resultant tissue pellets were resuspended in 1–3 mL of accutase (PAA Laboratories GmbH Yeovil, Somerset, UK) with 200 U/mL DNase1 (Sigma-Aldrich, Poole, Dorset, UK) and incubated at 37 °C with regular gentle trituration, for 10–30 min, before the addition of equal volumes of Advanced DMEM/F12 medium (Invitrogen Life Technologies), and final mechanical disaggregation. The cell suspensions were centrifuged at 110*g* for 5 min and the pelleted cells were resuspended in 2–3 mL differentiation medium with the following composition: Advanced DMEM/F12 (Invitrogen Life Technologies) adjusted to 1.8 mM CaCl_2_ (Sigma-Aldrich) and supplemented with 2 mM L-glutamine 1% penicillin/streptomycin (Invitrogen Life Technologies), 0.5 mM valproic acid (Sigma-Aldrich) and 2% NeuroBrew21 (with vitamin A, MACS Miltenyi Biotec, Bisley, Surrey, UK). Finally, neurons were plated onto 1 mg/mL poly-D-lysine (PDL, Sigma-Aldrich)-coated 13-mm glass coverslips (VWR International, Lutterworth, Leicestershire, UK) at a density of approximately ~ 1 × 10^5^ cells/coverslip and were cultured in differentiation medium for up to 16 days at 37 °C in a humidified atmosphere of 5% CO_2_/95% air. Differentiation medium was changed every 2–3 days. Striatal neurons were used for further experimentation from 1 day post-plate down (dpp) onwards.

### Human iPSC culture, differentiation and transfection

Two hiPSC lines were employed in this study. Firstly, the CS83iCTR33 (CTR33Qn1) line which was derived from an unaffected sibling of a patient with Huntington’s disease (HD), with genotyped *CAG* repeat lengths of 33 and 18 in the *HTT* gene; this line was reprogrammed using a non-integrating strategy and was developed as a ‘control’ line for a related study on Huntington’s disease hiPSC characterisation, similar to that already published for integrating HD hiPSC lines [25]. Secondly, the 34D6 line [2] was derived from a ‘control’ patient and re-programmed using integrating vectors, a kind gift of Prof. Siddharthan Chandran, University of Edinburgh.

hiPSC lines were cultured in the pluripotent state using mTeSR™1 (Stem Cell Technologies, Grenoble, France) on BD Matrigel-coated plates (BD Biosciences, Oxford, Oxon, UK), with dispase passaging following the manufacturer’s instructions (Stem Cell Technologies). hiPSCs were differentiated to neural progenitors using an in-house, patented differentiation protocol (PCT/GB2014/053064) previously described [37], that is similar to that described for hES cells [26]. To generate ventral forebrain neural progenitors, hiPSCs were plated onto Matrigel-coated glass coverslips and transferred into SLI medium (Advanced DMEM/F12, supplemented with 2 mM L-glutamine, 1% penicillin/streptomycin, 2% NeuroBrew21 (without vitamin A), 10 μM SB431542 (Abcam, Cambridge, Cambridgeshire, UK), 1 μM LDN 193189 (Stemgent, Cambridge, MA, USA) and 1.5 μM IWR1 (Tocris Bioscience, Abingdon, Oxon, UK); this point was designated as 0 dpp. Cells were cultured for 4 days in SLI medium with medium changed daily. At 5 dpp, cells were treated with 10 μM Y-27632 (Abcam) for 1 h prior to passage using accutase and replating on Matrigel-coated plates with a split ratio of 1:2. At 8 dpp, cells were passaged as above and the medium was switched from SLI to LI medium (Advanced DMEM/F12, supplemented with 2 mM L-glutamine, 1% penicillin/streptomycin, 2% NeuroBrew21 (without vitamin A), 250 nM LDN 193189 and 1.5 μM IWR1). The medium was changed daily until day 16 dpp, when neural progenitors were harvested by accutase (GE Healthcare Life Sciences, Little Chalfont, UK) treatment and centrifugation at 110*g*, and the cell pellets resuspended as a single cell suspension, and cultured on Matrigel-coated coverslips at a density of approximately 1 × 10^5^ cells/coverslip in SCM1 differentiation medium (Advanced DMEM/F12, supplemented with 2 mM L-glutamine, 1% penicillin/streptomycin, 2% NeuroBrew21 (with vitamin A), 2 μM PD0332991 (Tocris Bioscience), 10 μM DAPT (Tocris Bioscience), 10 ng/mL BDNF (MACS Miltenyi Biotec), 1 μM LM22A4 (Tocris Bioscience), 10 μM forskolin (Tocris Bioscience), 3 μM CHIR 99021 (Tocris Bioscience), 300 μM GABA (Tocris Bioscience), 1.8 mM/L CaCl_2_, 200 μM ascorbic acid (Sigma-Aldrich)). At 23 dpp, SCM1 medium was exchanged for SCM2 (1:1 Advanced DMEM/F12: Neurobasal A (Invitrogen Life Technologies), supplemented with 2 mM L-glutamine, 1% penicillin/streptomycin, 2% NeuroBrew21 (with vitamin A), 10 ng/mL BDNF, 1 μM LM22A4, 3 μM CHIR 99021, 1.8 mM/L CaCl_2_, 200 μM ascorbic acid) and cultured in SCM2 for up to 37 dpp with 50% medium changes every 3–4 days.

Sixteen dpp CTR33Qn1 and 34D6-hiPSC-derived neural progenitor cells were co-transfected with Kv7.2/7.3 cDNA plasmid concatemer [42] and pmax®GFP (Lonza, Cologne, Germany) at a ratio of 10:1 using the Amaxa Nucleofector human stem cell kit (Lonza), following the manufacturer’s protocols. As a control, a sham transfection was performed using the empty vector. After transfection, the cells were plated and cultured in SCM1 on PDL- and Matrigel-covered glass coverslips and, after incubation for 24 h, were transiently selected with 800 μg/mL G418 (Sigma-Aldrich). SCM1 was exchanged for SCM2 after 23 dpp and cells were cultured for up to 37 dpp.

### Electrophysiological recordings and analyses

Voltage and current recordings were made using conventional patch-clamp in the whole-cell configuration [15, 37]. The bath solution contained (in mM) 135 NaCl (Fisher Scientific UK Ltd, Loughborough, Leicestershire, UK), 5 KCl (Fisher), 1.2 MgCl_2_ (Sigma-Alrich), 1.25 CaCl_2_ (Sigma-Aldrich), 10 D-glucose (Fisher) and 5 N-2-hydroxyethylpiperazine-N′-2-ethanesulfonic acid (HEPES, VWR International); pH was adjusted to 7.4 using 5 M NaOH. The pipette solution contained (in mM) 117 KCl, 10 NaCl, 11 HEPES, 2 Na_2_-ATP (Sigma-Aldrich), 2 Na-GTP (Sigma-Aldrich), 1.2 Na_2_-phosphocreatine (Sigma-Aldrich), 2 MgCl_2_, 1 CaCl_2_ and 11 ethylene-glycol-tetra-acetic acid (EGTA, Fisher); pH was adjusted to 7.2 with KOH. All electrophysiological studies were performed using an Axopatch 200B amplifier and Digidata 1322A A/D interface (Axon Instruments, Forster City, CA, USA) at a controlled room temperature of 22 ± 0.5 °C. Recordings were digitised at 10 kHz and low-pass filtered at 2 Hz using an 8-pole Bessel filter. The patch-clamp data were analysed using Clampfit 9.0, Microsoft Excel and Microcal Origin 6.0 software.

Membrane potential (Vm) was recorded in current-clamp mode. Mean (± SEM) membrane potential values were plotted against dpp. Neurons were coded according to the type of sAP activity that they demonstrated, defined as follows: (1) spontaneous action potentials (Spontaneous, at least 1 excursion that overshoots 0 mV), (2) attempted spontaneous action potentials (Attempting, significant excursions from resting Vm that did not reach 0 mV) or (3) no action potentials (Quiet, no significant excursions from resting Vm). This then allowed neurons to be categorised into the three separate groups for further detailed analysis. Once Vm and spontaneous activity had been recorded, current was injected to hyperpolarize Vm to ca. − 70 mV before 1 s current injection steps were imposed (from − 10 to + 180 pA) in order to induce action potentials. Input resistance was measured from the voltage difference induced by the − 10-pA current step to record induced action potential activity, which was coded as none (no significant excursions from baseline during injection), attempting single (significant excursions from baseline that do not reach 0 mV), single (a single excursion that overshoots 0 mV), attempted train (several excursions but only 1 overshoots 0 mV) and train (several excursions, at least 2 of that overshoot 0 mV). Where induced action potential trains were recorded, a spike frequency analysis was performed. Input resistance was measured from the voltage difference induced by the − 10-pA current step. Spike analysis was performed on the first spike of an induced action potential train using Clampfit 9; threshold was determined as the peak of the 3rd differential of voltage with respect to time during the upstroke of the action potential, and all other parameters are as defined extensively elsewhere [1].

Na^+^ currents were recorded using a standard voltage-step protocol (holding potential of − 70 mV followed by 80 ms steps from − 120 to + 80 mV in increments of 10 mV). For Na^+^ current inactivation curves, cells were stepped for 200 ms to pre-pulse voltages of between − 120 and + 80 mV in 5 mV step increments before being stepped for 200 ms to the test potential of 0 mV. Cell capacitance and series resistance were measured and compensated; series resistance was compensated 60–90%. Pipette resistances were 8–10 MΩ when filled with the pipette solutions.

Conductances (*G*) for activation and inactivation were calculated by dividing current by the appropriate driving force, (*V*_c_ − *E*_Na_), where *V*_c_ = command potential, and *E*_Na_ = +66.7 mV. *G*/*G*_max_ was plotted against voltage and fitted with a Boltzmann equation using an iterative fitting routine:

$$ G/{G}_{\mathrm{max}}=1/\left[1+\exp \left({V}_{50}-{V}_{\mathrm{c}}\right)/h\right] $$

where *G*_max_ is the extrapolated maximum conductance, Va_50_ and Vi_50_ are the voltages corresponding to half the maximum conductance and *h* is the slope factor.

#### Multi-electrode arrays

CTR33Qn1 hiPSCs at 16 dpp were plated at a density of 150 × 10^3^ cells/well onto poly-D-lysine-coated, 24-well multi-electrode arrays (MEAs) and cultured sequentially using the differentiation protocol as described above and in the previous study [37]. At 21 dpp, the medium was switched for a HEPES-buffered physiological solution containing the following (in mM): 130 NaCl, 3 KCl, 1 MgCl_2_, 2 CaCl_2_, 10 D-glucose and 10 HEPES-NaOH at pH 7.4, and the MEAs were placed onto the temperature-controlled (37 °C) platform of a commercially available MEA workstation (Multichannel Systems, Reutlingen, Germany). Each well contained 12 electrodes, the activities of which were recorded simultaneously before, during and following manual addition of agonists, antagonists and toxins, as defined in the text and legends. Data were band-pass filtered between 100 and 2000 Hz and finally sampled at 20 kHz using the acquisition software provided with the device. During recordings, this software detected single unit activity online and live displays of raw data, spikes per second histograms, and detected spike waveforms were available. For analysis, raw data were converted to a HDF5 file format, which was imported into the MatLab environment in which custom-written analysis routines were coded. Spikes were redetected and sorted, and time stamps were extracted allowing the various plots shown here to be produced. Root mean square recording noise levels were ~ 1.7 μV. Spikes were detected using a threshold method (usually 5 SD of the mean) that detected either rapidly rising or falling events. Amplitudes of clearly resolvable spikes uncontaminated by significant levels of false detections varied between 10 and 100 μV. The major source of this amplitude variation is likely to be the physical distance of each detected neuron from the recording site. Typically, spontaneous spiking activity could be detected on multiple electrodes within a single well and in 24-well plates.

Gabazine hydrobromide (SR-95531) (Tocris Bioscience) at 5 μM was employed as a selective competitive antagonist of GABA_A_ receptors [37]. Retigabine dihydrochloride (LKT Laboratories Inc., St Paul, MN, USA) at 10 μM was employed as a selective opener of Kv7 channels [42]; XE991 (Sigma-Aldrich) at (10 μM) was used as a selective blocker of Kv7 channels [40].

All data are expressed as mean ± SEM. Statistical comparisons of the means were performed using 2-way AVOVA or Student’s *t* tests, as appropriate; differences were considered significant at *p* < 0.05.

### Human tissue collection, processing and data analysis

Adult motor cortical, caudate and putamen samples from 31- to 86-year-old donors were obtained from the Neurological Tissue Bank of the Biobank-Hospital Clínic (IDIBAPS) following the guidelines and approval of the local ethics committee (Hospital Clínic of Barcelona’s Clinical Research Ethics Committee). Total RNA was isolated using TRI Reagent (Sigma-Aldrich) following the manufacturer’s protocol. Human foetal tissue (CRL of 22–54 mm) ranging in age from 7 to 9 weeks post-conception was collected by donation of the products of elective termination of pregnancy, within the MRC- and Welsh Government-sponsored South Wales Initiative for Foetal Tissue Transplantation (‘SWIFT’) programme, with full ethical approval (02/4446). Tissue was collected directly into hibernation medium at 4 °C and transported to Cardiff University where it was dissected under category II culture conditions, according to a previously published protocol [19]. Cortex and striatal primordia were dissected in 0.9% saline solution with addition of 0.6% glucose (hospital pharmacy) and stored individually in TRIzol (Invitrogen Life Technologies) at − 80 °C until use. Total RNA was isolated using TRI Reagent (Sigma-Aldrich) or TRIzol following the manufacturer’s protocol. Then, 10 μL of total RNA at a concentration of 200 ng/μL (2 μg in total) for each sample was reverse-transcribed with random primers using High-Capacity RNA-to-cDNA Kit (Invitrogen Life Technologies); 10 μL of retro-transcription cocktail (2 μL of 10× RT buffer, 2 μL pf Random primers,1 μL of dNTP mix; 1 μL MultiScribe reverse transcriptase) was added to each sample (20 μL total volume). After gentle mixing, samples were incubated for 10 min at room temperature followed by 2 h at 37° C, 10 min on ice and 10 min at 75° C; 10 ng of cDNA was used to perform quantitative real-time PCR (qPCR). PrimeTime qPCR assays were used as recommended by the provider (IDT Technologies, Leuven, Belgium). RPL13A, B2M and HSP90 mRNA levels were used as reference genes. qPCR was carried out with Premix Ex Taq (Takara Clontech, Saint-Germain-en-Laye, France) in 6 μL of final volume using CFX384-C1000 Thermal Cycler equipment (Bio-Rad). Samples were run for 40 cycles (95 °C for 5 s, 60 °C for 20 s). Relative gene expression values were calculated using the 2^−∆∆Ct^ [22] using Bio-Rad CFX manager software (Bio-Rad, Madrid, Spain). All data are expressed as mean ± SEM. Data were analysed using GraphPad 4.02. Statistical comparisons of the means were performed using two-tailed, unpaired *t* test; differences were considered significant at *p* < 0.05.

### Mouse tissue collection and processing for microarrays and qPCR

C57/Bl6 mice (Charles River Laboratories, Les Oncins, France) were maintained in standard conditions with food and water ad libitum. All animal procedures were approved by local committees, in accordance with the European Communities Council Directive (86/609/EU). The day of pregnancy, determined by the first detection of a vaginal sperm plug in daily inspection, was considered embryonic day (E) 0.5. Briefly, brains were removed, frozen and embedded before sections were cut using a cryostat, and after cresyl violet staining, the two different zones of the lateral ganglionic eminence (LGE) germinal zone (GZ) and mantle zone (MZ) were microdissected using the Leica Laser Microdissection Microscope D7000. RNA extraction of the collected samples was carried out with the RNeasy Micro Kit (Qiagen). Microarrays were run using the Affymetrix MouseGene ST v1.1 chip to analyse 32 mRNA samples (5–4 samples per condition). To validate the dynamics of Kv7.2 (*KCNQ2*) and Kv7.3 (*KCNQ3*) gene expression during mouse embryonic development, mRNA levels of both channels were analysed by qPCR at different developmental stages (E12.5, E14.5, E16.5 and E18.5) and in the different striatal zones (LGE for E12.5, GZ and MZ for the other stages). Expression of mRNA encoding *KNCQ* channels in embryonic mouse striatal (mSTM) neurons was determined by qPCR (see above, for conditions). All data are expressed as mean ± SEM. Data were analysed using GraphPad 4.02. Statistical comparisons were made by two-way analysis of variance (ANOVA) followed by Bonferroni post-test. Values of *p* < 0.05 were considered statistically significant.

### Immunocytochemistry

Cells were fixed with 4% paraformaldehyde in PBS (Invitrogen Life Technologies) for 15 min at 37 °C. Cells were washed (3×) for 5 min, blocked and permeabilised using PBS containing 1% BSA (*w*/*v*) 0.1% Tween-20 for 1 h at room temperature before being incubated overnight at 4 °C in rabbit primary antibody solutions (anti-*KCNQ2* at 1:100 (Abcam, ab22897); anti-*KCNQ3* at 1:100 (Aviva Systems Biology Corporation, San Diego, CA, USA, OAAF00246)). Controls, where primary antibodies were omitted, were included for all experimental sets. After overnight incubation, the primary antibody solution was removed and the cells were washed for 5 min in PBS (3×) before incubation for 1 h with fluorophore-conjugated secondary antibody (Alexa Flour-488, goat anti-rabbit, Life Technologies) at room temperature in the dark. Nuclear staining employed Hoechst stain (Life Technologies) at 1:5000 in PBS. Coverslips were mounted in Fluoromount G (eBioscience, Hatfield, Hertfordshire, UK) and imaged using Olympus BX61 with SIS F-view charge-coupled device (CCD) camera and AnalySIS imaging software (Olympus, Southend-on-Sea, Essex, UK).

## Results

### Development of Vm and expression of Kv7.2 and Kv7.3 subunits during in vivo and in vitro maturation of mouse striatal neurons

**Fig. 1:**
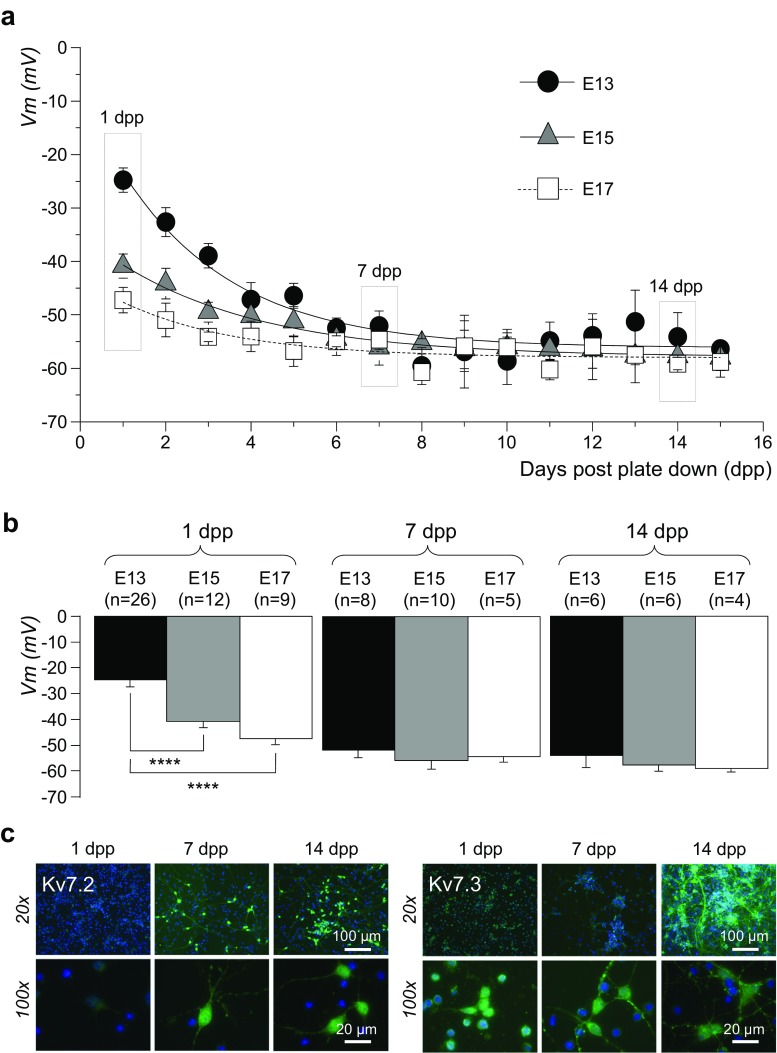
**Ontogeny of membrane potential and Kv7.2 and Kv7.3 channel protein expression in mouse striatal neurons isolated at embryonic stages E13, E15 and E17 and differentiated in** ***vitro***. **a** Exponential fits of membrane potential (Vm) changes in mouse striatal neurons isolated at embryonic stages E13 (black circles), E15 (grey triangles) and E17 (empty squares) and cultured for up to 15 days post-plate down (dpp). The dashed boxes represent the time windows for which the analyses in **b** have been derived. **b** Bar charts presenting means of Vm measured in mouse striatal neurons isolated at the embryonic stages indicated above each bar (E13 (black), E15 (grey) and E17 (white)) and assayed at 1 dpp (left), 7 dpp (centre) and 14 dpp (right). Number of observations shown in parentheses. Significant differences, determined by unpaired Student’s *t* test, are shown below the bars, where appropriate; ****p < 0.0001. **c** Kv7.2 (left panels, ×20 and ×100, as indicated) and Kv7.3 (right panels, ×20 and ×100, as indicated) protein expression by immunocytochemistry in E15 mouse striatal neurons during in vitro maturation at 1, 7 and 14 dpp

During the first dpp in vitro, mean Vm values of mouse striatal neurons isolated at embryonic stages E15 and E17 were significantly different (*p* < 0.001) from that of E13 neurons, but not significantly different from each other: E13 = − 24.8 ± 2.3 mV (*n* = 26), E15 = − 40.0 ± 2.2 mV (*n* = 12) and E17 = − 47.2 ± 2.4 mV (*n* = 9). These data suggest that the most important stage in the early development of a hyperpolarized Vm in vivo takes place between E13 and E15 (Fig. 1a, b). Over the next 6 dpp, mouse striatal neurons isolated at all three stages displayed a steady hyperpolarization to between − 50 and − 60 mV, with only modest hyperpolarization occurring thereafter up to 15 dpp (Fig. 1a, b). Mean Vm values of each group, at either 7 or 15 dpp, were not significantly different from each other (Fig. 1b).

Since one of the most important mechanisms responsible for setting and maintaining of Vm in the neurons is represented by Kv7 channels [3, 4, 18, 24], the expression of the Kv7.2 and Kv7.3 protein subunits was determined using immunocytochemistry in neurons isolated at E13 and cultured for up to 14 dpp. During in vitro differentiation and maturation, neurons became more complex and demonstrated a time-dependent expression of both Kv7.2 (Fig. 1c, left panel) and Kv7.3 (Fig. 1c, right panel) in processes and somata; no staining was observed in the negative control when the primary antibody was omitted (not shown).

### Ontogeny of *KCNQ* (Kv7) subunits in developing mouse and human striatum

**Fig. 2:**
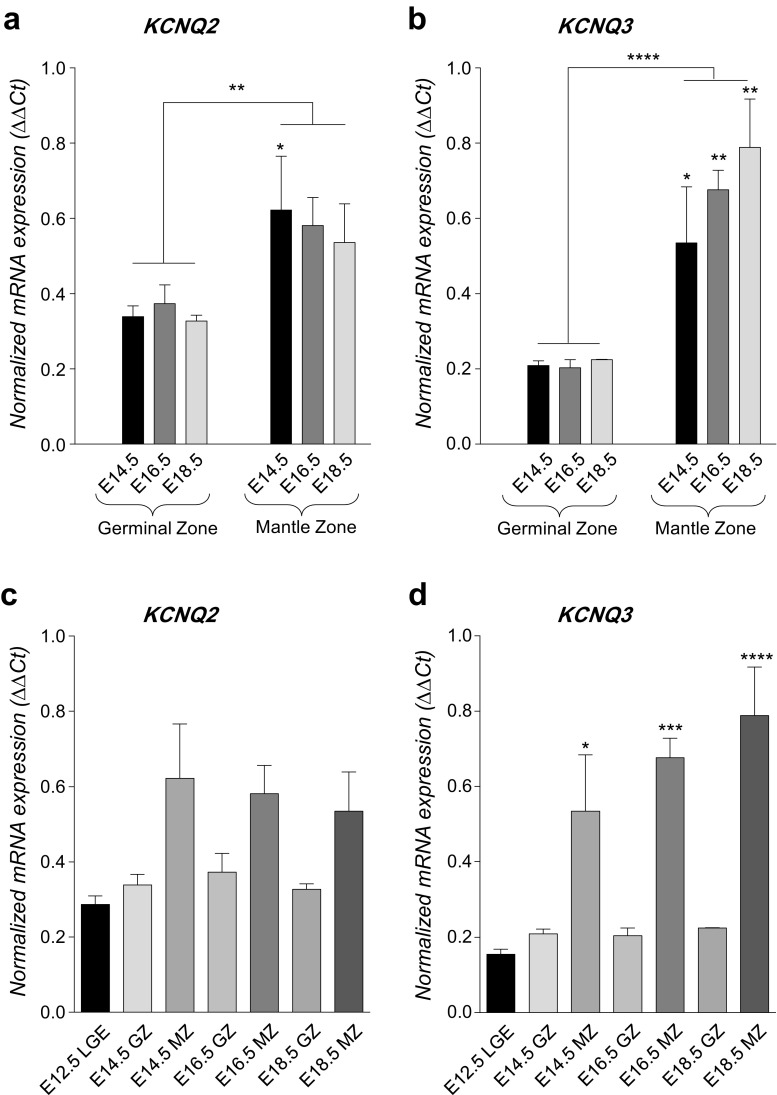
**Ontogeny of** ***KCNQ2*** **and** ***KCNQ3*** **mRNA expression in developing mouse striatum in** ***vivo***. **a** *KCNQ2* mRNA expression during mouse striatal development in vivo. In **a** and **b**, mRNA levels were determined in laser-microdissected mouse striatal regions by qPCR and were normalised to the house-keeping genes (18S/β-actin) using the ΔΔCt method. By two-way ANOVA, there was a significant difference between *KCNQ2* mRNA in mantle zone (MZ) and germinal zone (GZ), **p < 0.01, with a significant enrichment in MZ at E14.5, asterisk above the MZ bar, *p < 0.05. **b** *KCNQ3* mRNA expression during mouse striatal development in vivo. By two-way ANOVA, there were significant regional differences, ***p < 0.001, with significant enrichments in the MZ at E14.5, E16.5 and E18.5, asterisks above the MZ bars, *p < 0.05, **p < 0.01. **c** *KCNQ2* mRNA expression during mouse striatal development in vivo. For **c** and **d**, mRNA levels in laser-microdissected mouse striatal regions were determined by qPCR and were normalised to the house-keeping genes (18S/β-actin) using the ΔΔCt method. Data were analysed by one-way ANOVA with Tukey’s post hoc test, and showed that, compared to LGE, *KCNQ2* mRNA levels in each zone did not significantly alter during development in either zone. **d** *KCNQ3* mRNA expression during mouse striatal development in vivo. Data were analysed by one-way ANOVA with Tukey’s post hoc test, and showed that, compared to LGE, *KCNQ3* mRNA in the MZ was shown to increase steadily during development. *p < 0.05, ***p < 0.001, ****p < 0.0001

**Fig. 3:**
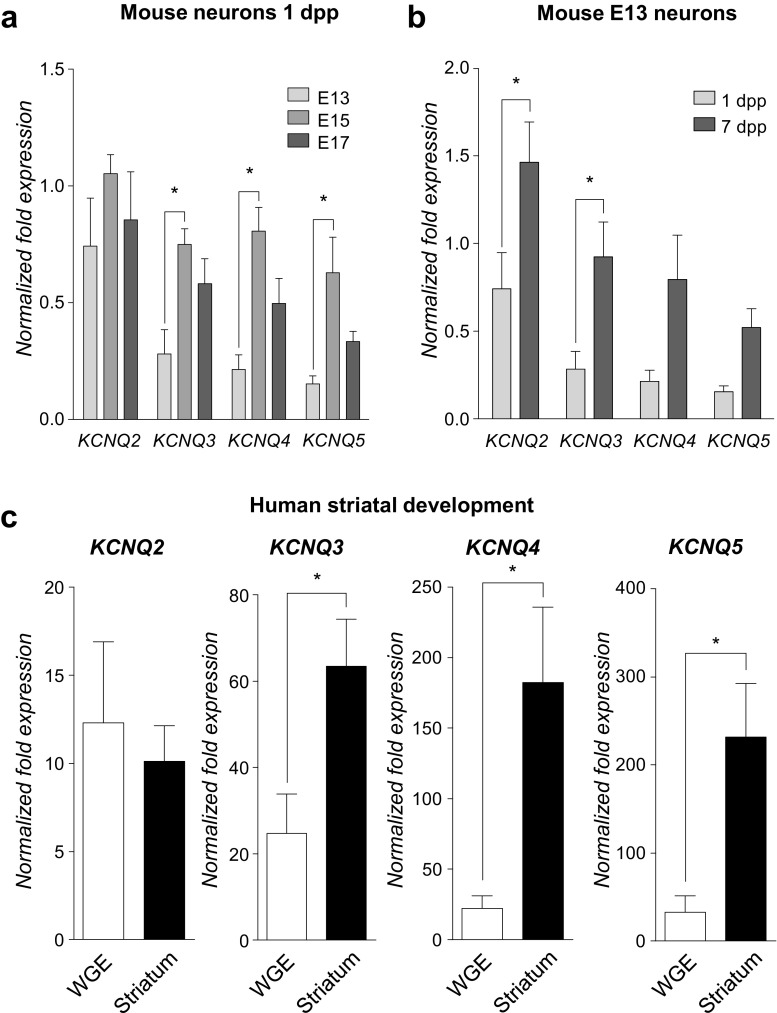
**Ontogeny of** ***KCNQ*** **mRNA expression in developing mouse and human striatum**. **a** *KCNQ* mRNA expression in mouse striatal neurons during development in vivo. mRNA levels were determined in mouse striatal neurons isolated at E13, E15 and E17 by qPCR and were normalised to the house-keeping genes (18S/β-actin) using the ΔΔCt method. By one-way ANOVA (Tukey’s post hoc), there were significant differences between KCNQ mRNA at E15 and E17 with its own control at E13, *p* < 0.05. **b** *KCNQ* mRNA expression in E13 mouse striatal neurons during in vitro differentiation and maturation. By one-way ANOVA (Tukey’s post hoc), there were significant differences of *KCNQ2* and *KCNQ3* mRNA between 1 and 7 dpp, *p* < 0.05. **c** *KCNQ* mRNA expression during human striatal development in vivo. mRNA levels in human striatal regions dissected from cryosections were determined by qPCR and were normalised to house-keeping genes (*RPL13A* and *HSP90AB1*) using the ΔΔCt method. By unpaired Student’s *t* test, there were significant differences between whole ganglionic eminence (WGE, 7–9 weeks post-conception) and adult striatum expression of *KCNQ3*, *KCNQ4* and *KCNQ5* mRNA, *p < 0.05

Having established that Vm and level of Kv7 channel subunit expression were subjects to developmental regulation, the ontogenies in vivo of the genes *KCNQ2* and *KCNQ3* (which encode Kv7.2 and Kv7.3 subunits, respectively) were determined in laser-microdissected mouse striata. Developmental time points of E12.5 (LGE) through E18.5 (GZ and MZ) were employed. For *KCNQ2* mRNA in vivo*,* two-way AVOVA highlighted an overall difference between GZ and MZ (*p* < 0.01), with a specific enrichment observed at E14.5 (*p* < 0.02, Fig. 2a). For *KCNQ3* mRNA, a similar analysis demonstrated regional differences (*p* < 0.001), with enrichments at E14.5 (*p* < 0.05), E16.5 (*p* < 0.01) and E18.5 (*p* < 0.01, Fig. 2b). Comparisons of mRNA levels in each zone with LGE demonstrated that the *KCNQ2* did not significantly alter during development in either zone (Fig. 2c). In contrast, *KCNQ3* steadily increased during development in the MZ (Fig. 2d). Thus, consistent with the electrophysiological observations, these data show that mRNA encoding both *KCNQ2* and *KCNQ3* becomes enriched in the post-mitotic MZ. Furthermore, they also suggest that the developmentally upregulated function of Kv7 channels is not a direct consequence of differential transcription of the *KCNQ2* gene but that of genes encoding other Kv7 subunits, such as *KCNQ3* (as shown in Fig. 2b, d), or even *KCNQ4* and *KCNQ5* (Fig. 3).

Further investigation of this idea was carried out by employing qPCR to quantify the expression of other *KCNQ* genes in mouse striatal neurons isolated between E13 and E17 and cultured for up to 7 DIV which, as a group, increased significantly (*p* < 0.001) between 1 and 7 dpp. Specifically, at 1 dpp, *KCNQ2* mRNA was expressed at similar levels across all developmental time points (Fig. 3a). However, *KCNQ3* mRNA (along with those for *KCNQ4* and *KCNQ5*) was upregulated between E13 and E15 (*p* < 0.05 for *KCNQ3* and *KCNQ5*, *p* < 0.01 for *KCNQ4*, Fig. 3a); thereafter, *KCNQ4* and *KCNQ5* expression declined whilst *KCNQ3* was sustained (Fig. 3a). During differentiation and maturation in vitro, a slightly different early pattern of mRNA expression was observed, with both *KCNQ2* and *KCNQ3* mRNA in mouse striatal neurons isolated at E13 increasing between 1 and 7 dpp (*p* < 0.05, Fig. 3b); mRNA encoding *KCNQ4* and *KCNQ5* demonstrated no significant changes during in vitro differentiation (Fig. 3b).

Finally, we sought to determine whether the overall pattern of *KCNQ*/Kv7 expression that was observed in mouse was reflective of the situation in human development. To this end, cryosections were cut from human foetal brain samples collected 7–9 weeks post-conception, when developing striatum is effectively the whole ganglionic eminence (WGE) and *KCNQ* mRNA levels compared with post-mortem adult striatum. *KCNQ2* mRNA was expressed at similar levels between the stages (Fig. 3c). Strikingly, and similar to the pattern seen in developing mouse striatum, *KCNQ3* was significantly enriched in the striatum, as were *KCNQ4* and *KCNQ5* (Fig. 3c). These data, which demonstrate the enrichment of subunits which constitute Kv7 channels in the post-mitotic neuronal compartments of the developing striatum of mouse and human in vivo, and the broadly similar pattern which is seen when mouse striatal neurons are differentiated in vitro, correlate well with the functional data showing developmentally regulated progressive hyperpolarization of Vm (see Fig. 1).

### Expression of *KCNQ* and Kv7 channel subunits during neuronal differentiation of hiPSCs

**Fig. 4:**
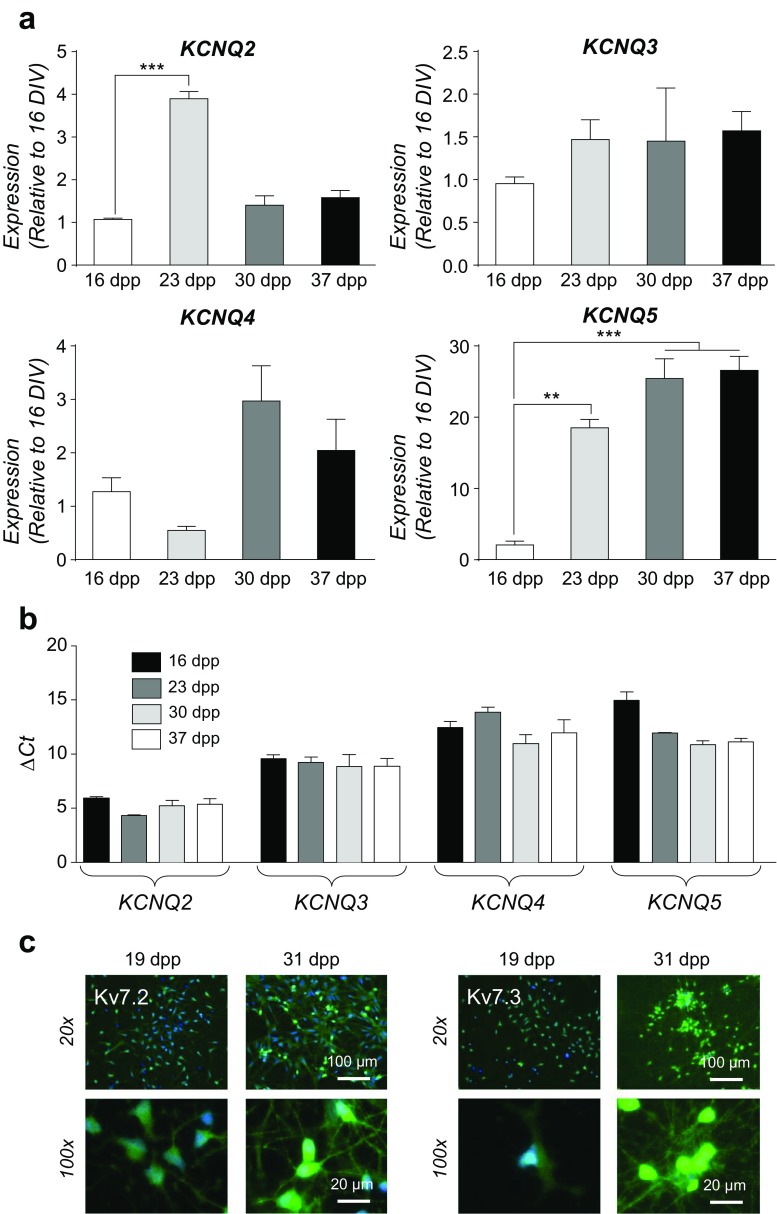
**Ontogeny of** ***KCNQ*** **mRNA and Kv7 channel protein expression during neuronal differentiation of CTR33Qn1 hiPSC-derived neurons**. **a** Ontogeny of *KCNQ2* (upper left), *KCNQ3* (upper right), *KCNQ4* (lower left) and *KCNQ5* (lower right) mRNA expression during neuronal differentiation of CTR33Qn1 hiPSC-derived neurons from pre-patterned forebrain progenitors (16 dpp) to maturation (37 dpp). mRNA levels were determined by qPCR, were normalised to the house-keeping genes (*B2M*, *RPL13A* and *HSP90AB1*) using the 2^−ΔΔCt^ method and expressed as a level relative to 16 dpp. Data were analyses by one-way AVOVA followed by Tukey’s post hoc test. Differences from 16 dpp are shown, **p < 0.01, ***p < 0.001. **b** Comparison of mean ∆Ct values of each *KCNQ* during CTR33Qn1 hiPSC neuronal differentiation. *KCNQ2* and *KCNQ3* values were significantly lower than *KCNQ4* or *KCNQ5*, indicating that the former are expressed at a higher level than the latter. Two-way ANOVA, *p* < 0.001. **c** Immunocytochemistry showing expression of Kv7.2 (left) and Kv7.3 (right) protein in CTR33Qn1 hiPSC-derived neurons at 19 dpp and 31 dpp during neuronal differentiation, at ×20 and ×100, as indicated

Although there have been observations that Kv7 channels contribute to the excitability of hiPSC-derived neurons [44], none have investigated directly the correlation between expression of Kv7 subunits, hyperpolarization of Vm and excitability during differentiation of hiPSCs, especially those pre-patterned to ventral forebrain precursors. Therefore, using the hiPSCs pre-patterned by an established technique of dual SMAD and Wnt inhibition [20, 26, 37], a detailed determination of both the expression of *KCNQ* mRNA and Kv7 subunits in these developing striatal-like neurons was performed using the control hiPSC line, CS83iCTR33Qn1 (CTR33Qn1). *KCNQ2* and *KCNQ5* mRNA both increased early (23 dpp), whilst *KCNQ3* and *KCNQ4* showed no significant differences during the differentiation protocol. Uniquely, *KCNQ5* remained elevated, whilst *KCNQ2* expression waned from 30 dpp onwards (Fig. 4a). With respect to absolute quantitation of *KCNQ* mRNA, *KCNQ2* and *KCNQ3* were expressed at significantly (*p* < 0.001) higher levels than *KNCQ4* and *KCNQ5* throughout the neuronal differentiation period (Fig. 4b), suggesting that these two subunits may contribute a quantitatively larger proportion to the outward current, a notion supported by the robust expression of Kv7.2 and Kv7.3 proteins during the differentiation protocol (Fig. 4c).

**Fig. 5:**
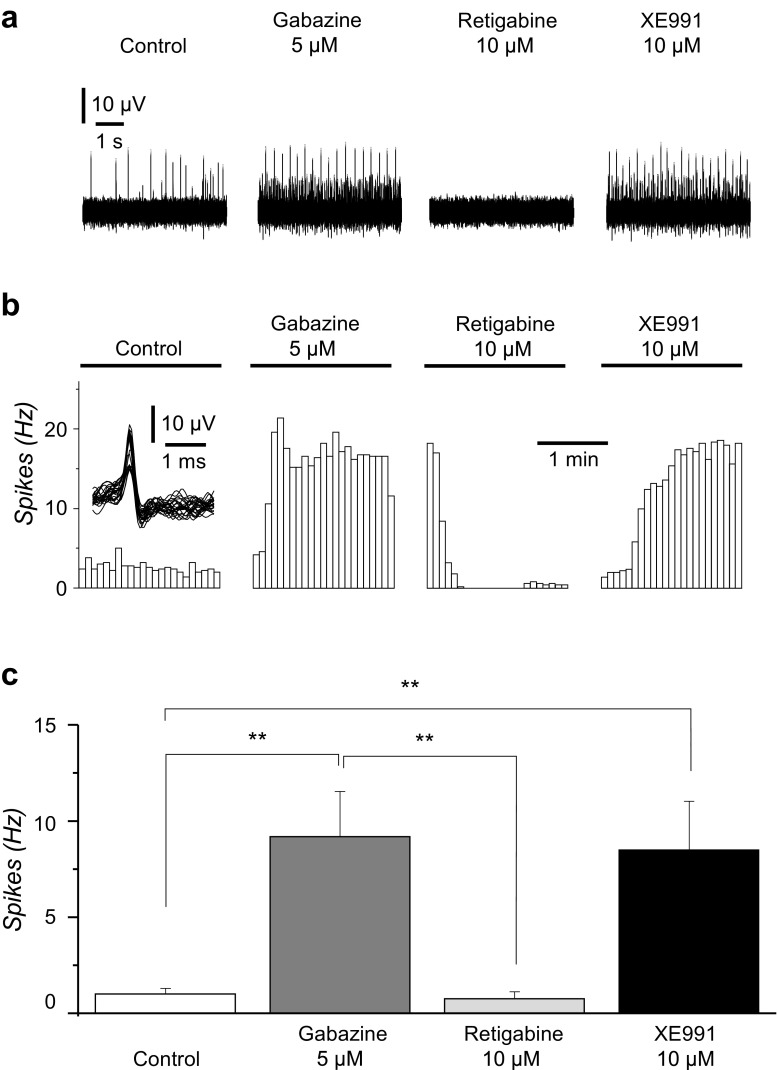
**Spontaneous unit responses recorded from CTR33Qn1 hiPSC-derived neurons plated in 24-well multi-electrode arrays (MEAs)**. **a** Exemplars of 5-s snapshots of raw data taken from one electrode (position 24) within one MEA well. The first recording (left) is in the absence of drugs. The subsequent recordings (right) are in the presence of GABA_A_ receptor-antagonist gabazine (5 μM), Kv7 channel opener retigabine (10 μM) and Kv7 channel selective blocker XE991 (10 μM). **b** Histograms of spike rate versus time for unit responses detected from individual MEA electrodes. Four consecutive 120-s recording epochs are shown from two electrodes, position 24 (top) and position 43 (bottom). The first epoch is in the absence of drugs. The inset shows 25 superimposed units detected during the first epoch. Drugs were applied immediately before the recording. Gabazine (5 μM) was applied initially to increase spontaneous activity of CTR33Qn1 hiPSC-derived neurons. Retigabine (10 μM) eliminated spiking and XE991 (10 μM) restored spiking to a rate similar to that observed before introduction of retigabine. **c** Bar charts presenting means of spike frequency ± SEM measured in CTR33Qn1 hiPSC-derived neurons displayed significant difference, determined by paired Student’s *t* test, in the presence of gabazine, retigabine and XE991 (**p < 0.01) compared to the control in the absence of drugs. A total of 11/12 electrodes detected units in this well and all responded to retigabine. In 7 instances, activity returned in the presence of XE991

Spontaneous neural network activity that CTR33Qn1 hiPSC-derived neurons developed between 30 and 37 dpp was recorded using MEAs. In the control, spontaneous neuronal firing was 1.0 ± 0.3 Hz (*n* = 12), and similar to our previous study [37], pharmacological inhibition of GABA_A_ receptors with gabazine (5 μM) resulted in a continuous and significant increase in the rate of neuronal firing to 9.2 ± 2.3 Hz (*n* = 12; vs. control *p* < 0.01) (Fig. 5). Retigabine (10 μM) suppressed gabazine induced firing to 0.8 ± 0.4 Hz (*n* = 12; *p* < 0.01), and Kv7 channel selective blocker XE991 (10 μM) re-established the firing to 8.5 ± 2.5 Hz (*n* = 12; vs. control *p* < 0.01) (Fig. 5).

### Functional maturation and forced expression of Kv7.2/7.3 channels in hiPSC-derived neurons

**Fig. 6:**
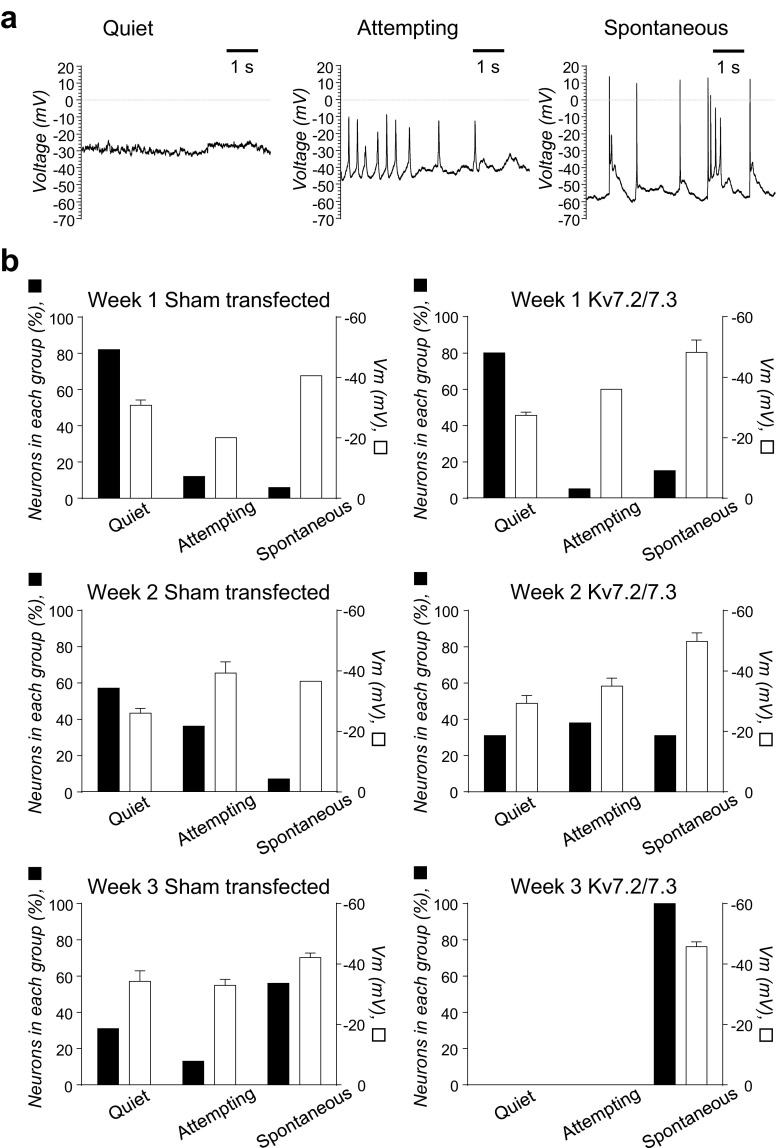
**Forced expression of Kv7.2/7.3 channel subunits facilitates functional maturation of in CTR33Qn1 hiPSC-derived neurons**. **a** Exemplar traces of current-clamp recordings (injected current = 0 pA) for CTR33Qn1 hiPSC-derived neurons exemplifying the model activity of no activity (Quiet), attempting activity (Attempting) or spontaneous activity (Spontaneous). **b** Bar graphs showing percentage of CTR33Qn1 hiPSC-derived neurons exhibiting no activity (Quiet), attempting activity (Attempting) or spontaneous activity (Spontaneous) (black bars, left *y*-axis) and their mean Vm (open bars, right *y*-axis) in sham-transfected (left panels) and Kv7.2/7.3 co-transfected CTR33Qn1 hiPSC-derived neurons (right panels) following 1, 2 and 3 weeks of in vitro neuronal differentiation

Based on the observations that *KCNQ* genes became highly and differentially expressed in mouse striatum, human striatum and hiPSC during in vivo and in vitro differentiation, the effects of forced co-expression of the two subunits, Kv7.2 and Kv7.3, known to constitute heteromeric Kv7 channel, were investigated. This was carried out in an attempt to assess whether such an experimental manoeuvre might accelerate and/or synchronise the functional maturation of hiPSC-derived neurons in order to provide a rational basis for enhancing current neuronal differentiation protocols. In these experiments, we measured the individual Vm values and ongoing electrical activity. For the latter, we classified hiPSC-derived neurons in the same way we did in the previous study [37] as showing absence of any activity (‘Quiet’), only sub-threshold transient depolarizing events (‘Attempting’ activity) and activity with overshooting action potentials (true ‘Spontaneous’ activity, Fig. 6a). Sham-transfected, pre-patterned, CTR33Qn1-derived neurons showed rather little spontaneous action potential generation during the first 2 weeks of neuronal differentiation (6% (1/17) at week 1 and 7% (1/14) at week 2); this had risen to 56% (9/16) by week 3 (Fig. 6b, Table 1). Forced co-expression of Kv7.2/7.3 resulted in a dramatic acceleration of maturation, such that the proportion of spontaneously active neurons was augmented to 17% (3/18) at week 1, 31% (5/16) at week 2 and, most strikingly, 100% (12/12) at week 3 (Fig. 6b, Table 1). These differences correlated well with resting Vm.

**Table 1:** Proportions and percentages of sham-transfected (left) and forced Kv7.2/7.3 co-expressing (+ Kv7.2/7.3, right) CTR33Qn1-derived neurons which demonstrated each of the different types of spontaneous action potentials (Quiet, Attempting or Spontaneous) during weeks 1 (upper), 2 (middle) and 3 (lower) of neuronal differentiation post-plate down in vitro

Week	Activity type	Sham transfected		+ Kv7.2/7.3	
		Proportion	%	Proportion	%
1	Quiet	14/17	82	14/18	78
	Attempting	2/17	12	1/18	5
	Spontaneous	1/17	6	3/18	17
2	Quiet	8/14	57	5/16	31
	Attempting	5/14	36	6/16	38
	Spontaneous	1/14	7	5/16	31
3	Quiet	5/16	31	0/12	0
	Attempting	2/16	13	0/12	0
	Spontaneous	9/16	56	12/12	100

This enhanced functional maturation could have resulted from Kv7-dependent hyperpolarization or from other changes in the resting or excitable membrane properties of the neurons. To test this, we measured the initial resting Vm, then neutralised any difference in potential by imposing a steady hyperpolarization to − 70 mV, followed by 100-ms current injection in order to induce action potentials; the effects of dpp and Kv7.2/7.3 forced co-expression were determined statistically using two-way ANOVA. For Vm, there were significant effects of both Kv7.2/7.3 overexpression (*p* < 0.0001) and weeks in vitro (*p* < 0.01). Indeed, by week 3, Vm of the sham-transfected neurons had hyperpolarized to − 38.5 ± 1.7 (*n* = 16, *p* < 0.05), whilst that of the Kv7.2/7.3 overexpressing neurons had hyperpolarized to − 45.5 ± 1.7 mV (*n* = 11, *p* < 0.05); this was 7 mV more negative than 3-week sham-transfected neurons (*p* < 0.01). For Rin, there were significant effects only of Kv7.2/7.3 overexpression (*p* < 0.02), presumably reflecting enhanced M-current development; see further below and Table 2.

**Table 2:** Analysis of passive and active parameters of induced action potentials from sham-transfected (left) and forced Kv7.2/7.3 co-expressing (right) CTR33Qn1-derived neurons during 1 (upper), 2 (middle) and 3 (lower) weeks of neuronal differentiation post-plate down in vitro

Week		Action potential parameters	Sham transfected			+ Kv7.2/7.3		
			Mean	SEM	*n*	Mean	SEM	*n*
1	Passive	Vm (mV)	− 30.2	1.8	17	− 30.9	2.0	20
		Rin (GΩ)	1.65	0.14	16	1.12	0.11	17
		Cp (pF)	14.1	2.0	13	13.7	1.4	15
	Spike analysis	Threshold (mV)	− 30.6	2.4	16	− 27.9	1.7	16
		Overshoot (mV)	29.0	4.5	16	25.2	4.0	16
		Afterhyperpolarization (mV)	− 57.0	4.3	16	− 54.3	2.7	16
		Amplitude (mV)	86.0	6.4	16	79.5	5.2	16
		Depolarization rate (V/s)	70.0	12.2	16	62.3	9.9	16
		Repolarization rate (V/s)	− 49.6	9.5	16	− 38.6	6.7	16
		Half-width (ms)	2.9	0.4	16	3.1	0.3	16
2	Passive	Vm (mV)	− 31.4	2.3	14	− 37.8	2.6	16
		Rin (GΩ)	1.36	0.19	12	0.88	0.12	11
		Cp (pF)	10.9	2.8	8	17.5	4.2	9
	Spike analysis	Threshold (mV)	− 38.0	3.0	12	− 30.9	3.2	11
		Overshoot (mV)	33.2	6.9	12	37.4	6.9	11
		Afterhyperpolarization (mV)	− 64.3	5.7	12	− 56.1	2.4	11
		Amplitude (mV)	97.5	11.6	12	93.5	6.7	11
		Depolarization rate (V/s)	83.0	14.0	12	94.2	18.4	11
		Repolarization rate (V/s)	− 56.2	12.6	12	− 45.8	8.8	11
		Half-width (ms)	3.0	0.5	12	2.8	0.3	11
3	Passive	Vm (mV)	− 38.5**	1.7	16	− 45.5**	1.7	11
		Rin (GΩ)	0.79	0.06	12	0.61	0.05	10
		Cp (pF)	17.1	2.8	11	16.3	2.5	10
	Spike analysis	Threshold (mV)	− 33.0	2.5	12	− 32.4	3.9	10
		Overshoot (mV)	34.19	5.1	12	42.2	3.8	10
		Afterhyperpolarization (mV)	− 56.0	5.2	12	− 60.7	2.9	10
		Amplitude (mV)	90.2	7.8	12	102.9	3.3	10
		Depolarization rate (V/s)	84.8	10.5	12	104.7	10.0	10
		Repolarization rate (V/s)	− 40.9	5.0	12	− 49.4	6.4	10
		Half-width (ms)	3.0	0.6	12	2.9	0.6	10

*Vm* membrane potential, *Rin* input resistance, *Cp* whole-cell capacitance

**Significantly different from each other at *p* < 0.01

The majority of CTR33Qn1-derived neurons, including those at week 1, demonstrated robust induced action potentials following an imposed depolarization from − 70 mV, with a tendency towards more action potential trains as differentiation proceeded (Table 3). However, there were no significant effects of either weeks in vitro or Kv7.2/7.3 overexpression on any of the induced action potential parameters (threshold, overshoot, peak amplitude, afterhyperpolarization, peak rates of depolarization and repolarization or half-width). Hence, the simplest explanation for the increased spontaneous activity at week 3 and its amplification on overexpressing Kv7.2/7.3 channels is that it results directly from the membrane hyperpolarization, rather than from other changes in resting or excitable properties.

**Table 3:** Proportion (%) of sham-transfected (left) and forced Kv7.2/7.3 co-expressing (right) CTR33Qn1-derived neurons which demonstrated each of the different types of induced action potentials during weeks 1 (upper), 2 (middle) and 3 (lower) weeks of neuronal differentiation post-plate down in vitro

Week	Induced action potential type	Sham transfected		+ Kv7.2/7.3	
		Proportion	%	Proportion	%
1	None	1/17	6	0/16	0
	Attempting single	0/17	0	0/16	0
	Single	5/17	29	4/16	25
	Attempting train	1/17	6	3/16	19
	Train	10/17	59	9/16	56
2	None	0/12	0	0/11	0
	Attempting single	0/12	0	0/11	0
	Single	3/12	25	2/11	18
	Attempting train	1/12	8	2/11	18
	Train	8/12	67	7/11	64
3	None	0/12	0	0/11	0
	Attempting single	0/12	0	0/11	0
	Single	3/12	25	0/11	0
	Attempting train	0/12	0	0/11	0
	Train	9/12	75	0/11	100

None = no significant voltage excursions from baseline; Attempting single = voltage excursions which do not overshoot 0 mV; Single = one excursion only, but which overshoots 0 mV; Attempting train = several excursions, but only one which overshoots 0 mV; Train = several excursions, with more than one which overshoots 0 mV

**Fig. 7:**
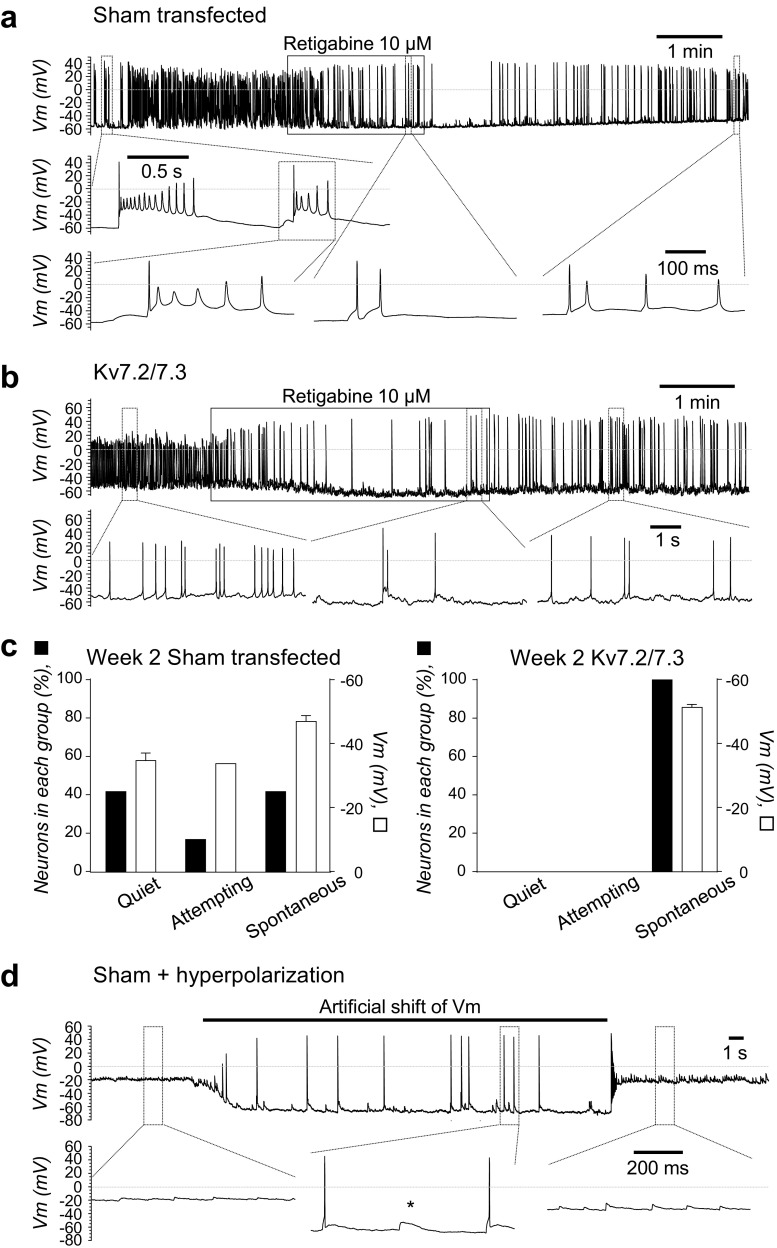
**Forced expression of Kv7.2/7.3 channels facilitates functional maturation of 34D6 hiPSC-derived neurons**. **a** Pharmacological identification of Kv7.2/7.3 channels (retigabine-activated) in an exemplar (injected current = 0 pA), sham-transfected, spontaneously active 34D6 hiPSC-derived neuron following 2 weeks of in vitro neuronal differentiation. Vm was measured in current-clamp mode of the whole-cell patch-clamp configuration in the absence and presence of 10 μM retigabine (indicated above the main trace by a transparent box). Below the trace are extracted action potential events shown on a fester time base. **b** Pharmacological identification of Kv7.2/7.3 channels (retigabine-activated) in an exemplar (injected current = 0 pA), Kv7.2/7.3 co-transfected, spontaneously active 34D6 hiPSC-derived neuron following 2 weeks of in vitro neuronal differentiation. Recording and display parameters as in **a**. **c** Bar graphs showing percentage of 34D6 hiPSC-derived neurons exhibiting no activity (Quiet), attempting activity (Attempting) or spontaneous activity (Spontaneous) (black bars, left *y*-axis) and their mean Vm (open bars, right *y*-axis) in sham-transfected (left panel) and Kv7.2/7.3 co-transfected 34D6 hiPSC-derived neurons (right panel) following 2 weeks of in vitro neuronal differentiation. **d** Neuronal activity in an exemplar in untransfected quiet 34d6 hiPSC-derived neuron following 2 weeks of differentiation evoked by artificial current injection to bring Vm below − 50 mV (indicated by a black bar above the main trace). Recording and display parameters as in **a**

In order to validate these data, experiments were performed in an unrelated hiPCS line, 34D6. After 2 weeks of differentiation, in those pre-patterned 34D6-derived neurons which exhibited spontaneous activity, application of 10 μM of the Kv7 channel opener, retigabine, resulted in reversible hyperpolarizing responses which were significantly larger in the Kv7.2/7.3 (− 7.7 ± 2.0 mV, *n* = 3) than in the sham group (− 5.4 ± 0.8 mV, *n* = 3, *p* < 0.03) and evoked a concomitantly more pronounced attenuation in neuronal firing rate Fig. 7a, b). At this point of neuronal differentiation, sham-transfected 34D6-derived neurons as a group exhibited a mean Vm of − 39.9 ± 2.2 (*n* = 12). Less than half (5/12) of this population, with a mean membrane potential of − 47.2 ± 1.9 mV, showed full spontaneous action potential activity (Fig. 7c). Of the remainder, 2/12 (− 31.5 and − 36.4 mV) showed some sub-threshold (‘Attempting’) activity, whilst 5/12 (mean Vm − 34.9 ± 2.9 mV) appeared totally quiet (Fig. 7c). Strikingly, forced co-expression of Kv7.2/7.3 resulted in significant hyperpolarization to a mean Vm of − 51.3 ± 1.1 mV (*n* = 11, *p* < 0.001), and now all of the cells showed full spontaneous action potential activity (Fig. 7b, c). To underscore the importance of a hyperpolarised Vm to the functional maturation of the neuronal network and gain further insight into its mechanism of generation, activity was measured during artificial hyperpolarisation by current injection. In those sham-transfected neurons which did not exhibit spontaneous activity (‘Quiet’), hyperpolarization of their Vm to − 70 mV resulted in all neurons (11/11 neurons) firing repetitively, as exemplified in Fig. 7d. Thus, these neurons clearly had the potential to generate spontaneous activity yet lacked the mechanism required to bring Vm to a level sufficiently hyperpolarized to remove Na^+^ channel inactivation; one such mechanism could well be the regulated expression of Kv7 channels.

**Fig. 8:**
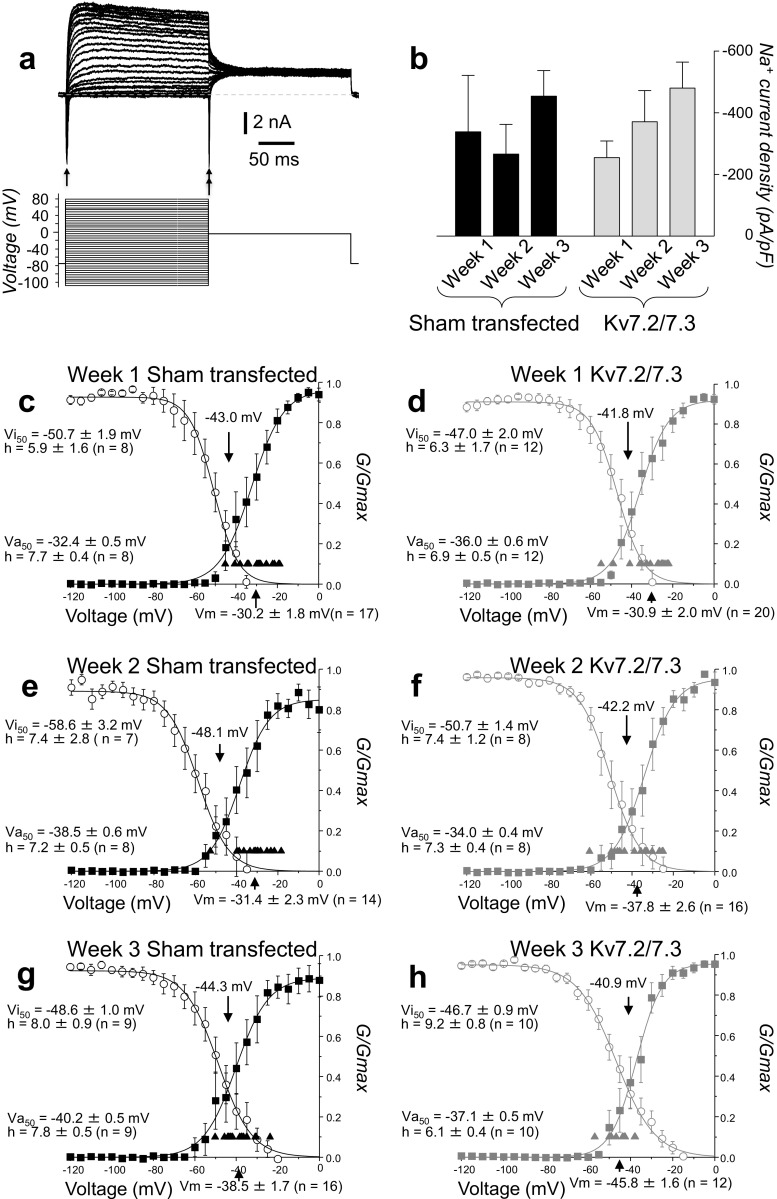
**Effect of forced expression of Kv7.2/7.3 channels on voltage-gated Na**^+^ **current activation and inactivation characteristics of CTR33Qn1 hiPSC-derived neurons**. **a** Exemplar family of whole-cell currents (upper) during the activation/inactivation voltage-clamp step protocol (lower). Peak Na^+^ current activation and inactivation levels are shown by the single- and double-headed arrows, respectively. **b** Mean peak ± SEM Na^+^ current densities measured from the peak of the inward currents exemplified in **a**. Data from sham-transfected week 1 (*n* = 8), week 2 (*n* = 7) and week 3 (*n* = 9) are shown by black bars. Data from Kv7.2/7.3 co-transfection week 1 (*n* = 12), week 2 (*n* = 8) and week 3 (*n* = 10) are shown by grey bars. **c**–**h** Mean activation and inactivation curves of whole-cell Na^+^ currents recorded in CTR33Qn1-dervied neurons cultured in vitro for 1 (**c**, **d**), 2 (**e**, **f**) and 3 weeks (**g**, **h**) following sham transfection (**c**, **e**, **g**, black symbols and lines) or forced co-expression of Kv7.2/7.3 (**d**, **f**, **h**, grey symbols and lines). Activation curves are depicted by the squares and inactivation curves are shown by the circles. On each panel, also shown are individual Vm values (triangles) and mean Vm values (arrow on *abscissa*). Voltages of half maximal action (Va_50_) and half maximal inactivation (Vi_50_) are also indicated, along with *h* factors, mean crossing points (downward arrows) and number of cells recorded for each group (*n*)

To address this point further, we recorded the Na^+^ currents during voltage-clamp in samples of sham-transfected and Kv7.2/7.3 overexpressing CTR33Qn1-derived neurons at weeks 1, 2 and 3 in culture (Fig. 8a). By two-way ANOVA, there were no significant effects of weeks in vitro or Kv7.2/7.3 overexpression (Fig. 8b). However, the progressive hyperpolarization, which occurred during differentiation, and which was enhanced by over co-expression of the Kv7.2/7.3 channels, brought resting Vm values within the range of overlap between the Na^+^ activation and inactivation curves (Fig. 8c–h), thus removing enough of the steady-state inactivation to facilitate action potential generation.

## Discussion

The role of ion channels in neurogenesis and stem cell development has been quite extensively studied. Evidence has been provided for a role of Ca_V_3.1 voltage-gated L-type Ca^2+^ channels in the differentiation of neural stem/progenitor cells [9] and of Kv3.1 K^+^ channels in the proliferation and neuronal differentiation of adult neural precursor cells [43]. Expression of Kv7.4 channels was recently reported in neurons derived from embryonic stem cells [28], and a potential role of Kv7.2/7.3 channels in differentiation, synaptogenesis and synaptic function of mouse hippocampal and embryonic stem cell-derived neurons has been indicated [44].

In the present study, we explored the role of Kv7 (Kv7.2/7.3) channels in the development and neuronal differentiation in mouse striatum and in the functional maturation of hiPSC-derived neurons. In the striatal neurons, we found a close correlation between the hyperpolarization of Vm, the expression of Kv7 channel subunit proteins both in vivo, between embryonic stages E13 and E17, and subsequently when cultured in vitro. Crucially, expression of mRNA encoding Kv7 subunits in the human developing striatum demonstrated a very similar ontogeny. Likewise, in the hiPSC-derived neurons, we observed a comparable relation between Kv7 channel expression and the development of normal excitable activity. In this latter case, at least, this relationship is likely to have been causal because the development of excitability was enhanced by overexpression of the Kv7.2/Kv7.3 heteromeric channels.

In addition to the increased overall Kv7 channel expression, another factor which might favour the increasing influence of the Kv7 channels in the maturation of the mouse MSNs could be the progressive switch from homomeric Kv7.2 to heteromeric Kv7.2/7.3 channels as suggested by the qPCR experiments. Since the heteromer has a higher affinity for the membrane phospholipid phosphatidylinositol-4,5-bisphosphate (PIP_2_) [21, 36], which is required for Kv7 channel opening, this itself might lead to a progressively larger current and increased membrane potential during development. In the hiPSC-derived neurons, the switch may be towards more Kv7.5 (*KCNQ5*), a subunit highly enriched in the adult striatum and which is a robust marker for human MSNs, as described in the Allen Human Brain Atlas (http://human.brain-map.org.).

The Kv7-driven membrane hyperpolarization would itself favour the expression of excitable properties, as indicated by the increased spontaneous activity of the developing hiPSC-derived neurons during a brief imposed hyperpolarization. Mechanistically, the key effect of developmental or imposed upregulation of the Kv7 channels was to bring the resting membrane potential within the optimal range for Na^+^ channel activation where activation and inactivation parameters overlap (and hence favour spontaneous action potential firing), without any change in the amplitude or kinetic parameters of the Na^+^ current or action potential themselves. However, further effects on the developmental process itself might well result from, for example, increased Ca^2+^ entry through co-activated voltage-gated Ca^2+^channels [9, 29] or an increased gradient for Ca^2+^ influx via other mechanisms. Further, K^+^ channels are known to be involved in the proliferation of non-excitable cells such as T lymphocytes [10] and a variety of tumour cells [16], through mechanisms that are not yet entirely clear; and Kv7.4 channels also appear to regulate skeletal muscle development [17].

The results presented herein show that an adequate expression of Kv7 channels in striatal and hiPSC-derived neurons is vital for functional maturation*,* demonstrating their crucial role in the normal development and maturation of striatal neurons and of hiPSC-derived striatal neurons. This knowledge can now be harnessed and applied to any pre-patterned neuronal precursor to synchronise and accelerate neuronal differentiation in order to generate meaningful, disease-relevant neurons for high-throughput screening and drug discovery for pathologies characterised by neural loss or degeneration.
